# Translating molecular medicine into clinical tools: doomed to fail by neglecting basic preanalytical principles

**DOI:** 10.1186/1479-5876-7-87

**Published:** 2009-10-14

**Authors:** Klaus Jung, Ferdinando Mannello, Michael Lein

**Affiliations:** 1 Department of Urology, Charité - Universitätsmedizin Berlin, Campus Mitte, Schumannstr. 20/21, 10117 Berlin, Germany; 2 Berlin Institute for Urologic Research, Berlin, Germany; 3 Department of Biomolecular Sciences, Section of Clinical Biochemistry, University "Carlo Bo", Urbino, Italy

## Abstract

This commentary discusses a study on measurements of matrix metalloproteinase 9 (MMP-9) in serum of pseudoxanthoma elasticum patients recently published in Journal of Molecular Medicine. This study can be considered the typical "obstacle" to effective translational medicine as previously documented in *JTM *journal. Although serum has been frequently proven as inappropriate sample for determining numerous circulating MMPs, among them MMP-9, there are over and over again studies, as in this case, that measure MMP-9 in serum. Comparative measurements in serum and plasma samples demonstrated higher concentrations for MMP-9 in serum due to the additional release from leukocytes and platelets following the coagulation/fibrinolysis process. From this example it can be concluded that translating basic research discoveries into clinical tools needs a more intensive exchange between basic biomedical research and clinical scientists already in an early stage. Otherwise a lost of translation, as discussed in *JTM *journal, seems to be inevitable.

## Commentary

Diekmann et al. [1] recently reported data in the Journal of Molecular Medicine on the increased serum concentrations of circulating matrix metalloproteinases 2 and 9 (MMP-2; MMP-9) in patients suffering from pseudoxanthoma elasticum. This genetic disorder, caused by mutations in the transporter gene *ABCC6*, is characterized by alterations in the extracellular matrix, especially in the skin, retina, and the vascular system. The authors reported that MMP-9 in serum was found both in male and female patients about 2.5-times higher than that in healthy controls, whereas MMP-2 was elevated only in female patients. On this basis, the authors hypothesized that the development of the symptoms of pseudoxanthoma elasticum could be attributed to the action of MMPs, since these enzymes are well known to be involved in the initial step of damage and/or the following remodelling, repairing processes of extracellular matrix [2]. The authors concluded that the measurement of serum MMP-2 and MMP-9 could be applied for non-invasive monitoring of matrix-degradative processes in pseudoxanthoma elasticum. In this respect, the use of MMP-2 and MMP-9 as surrogate biomarkers suggested by Diekmann et al. [1] may be appreciated as a nice example of translational medicine, defined as "*the transfer of new understandings of disease mechanisms gained in the laboratory into the development of new methods for diagnosis, therapy, and prevention and their first testing in humans*" [3] or "*effective translation of the new knowledge, mechanisms, and techniques generated by advances in basic science research into new approaches for prevention, diagnosis, and treatment of disease ....for improving health*" [4].

The study of Diekmann et al. [1] deals with an interesting topic and shows the potential of basic science discovery to improve clinical medicine. However, a closer and accurate re-examination of this "bench-to-bedside" example manifests that Diekmann et al. [1] have neglected the opposite "bedside-to-bench" effort of translational medicine as second part of its "two-way road" principle [5]. According to growing literature evidence demonstrating that blood sampling strongly influences the measurement and recovery of "true" circulating matrix metalloproteinases (MMPs) and their tissue inhibitors (TIMPs), we would like to draw attention on the preanalytical impact of blood collection/handling methods in order to limit technical pitfalls that may lead to misinterpretations. In particular, the authors did not consider that serum was demonstrated as inappropriate sample for measuring circulating MMP-9. Noteworthy, the misuse of serum as sample for determining circulating MMP-9 was frequently considered inadequate, both in clinical and biochemical/analytical journals [6-11]. It was additionally pointed out that technical details of sampling and handling procedures (like the time between venipuncture and centrifugation of blood samples as well as the use of different anticoagulants) must be taken into consideration with more attention and have to be reported due to their known crucial influence on the concentrations and activation/inhibition patterns of MMP-9 [12,13]. Thus, the fundamental significance of blood processing as important preanalytical determinant of accurate measurements of really circulating MMPs in peripheral blood, especially for MMP-9, has been clearly overlooked by Diekmann et al. [1]. It is a typical example that may be considered as of one of the significant "obstacles" to effective translational medicine contributing to the "lost of translation" as well documented in *JTM *journal [14].

**Figure 1:**
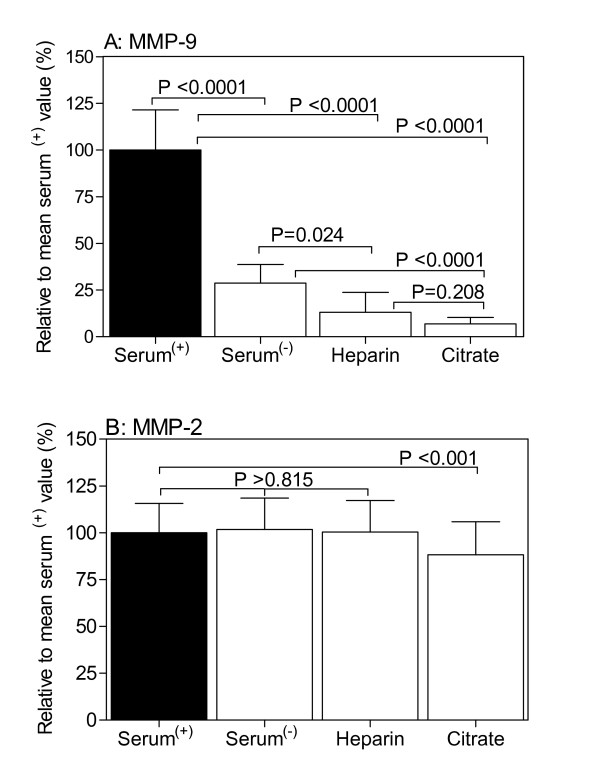
**Effect of blood sampling on MMP-9 (A) and MMP-2 (B) concentration in serum and plasma**. Values are given as mean values and their 95% confidence intervals relative to the mean value (=100%) of concentrations measured in serum^(+) ^samples from ten healthy adults. Statistical analyses were carried out by Student's *t*-test of paired data.

To highlight the role and effects of preanalytical conditions, we summarized in Figure 1 some of our own data of MMP-2 and MMP-9 measurements in serum and plasma samples collected under different conditions [15]. Briefly, from 10 healthy adults (all with normal leukocyte count and profile), venous blood samples were simultaneously collected in plastic tubes (Monovette Systems, Sarstedt AG, Nümbrecht, Germany). All subjects, informed about the objectives of the study, participated on a voluntary basis and provided informed consent. Tubes either without additives or with kaolin-coated granulate as clot activator were used to prepare native serum (serum^(-)^) or serum after enhanced coagulation (serum^(+)^), respectively; tubes with lithium heparin or sodium citrate were used to collect plasma samples. The blood specimens were centrifuged within 30 min after venipuncture at 1600 × g and 4°C for 15 min and the supernatants were carefully removed and stored at -80°C until analysis. MMPs were measured in duplicates with the Fluorokine MultiAnalyte Profiling assay system (R&D Systems, Minneapolis, MN, USA) on a Luminex 100 Bioanalyzer (Luminex Corp., Austin, TX, USA). The MMP assays detect, according to manufacturer's instructions, the corresponding pro-, mature, and tissue inhibitor of metalloproteinase (TIMP)-1-complexed MMPs. With regard to the measurements of MMPs in the different types of samples, the percentage analytical coefficients of variation calculated from the duplicate values were between 5.9% and 8.9% for MMP-2 and 4.1% and 8.4% for MMP-9, respectively.

Figure 1A shows that higher MMP-9 concentrations were found in serum in comparison with plasma samples. Moreover, the highest values of MMP-9 were observed in serum^(+) ^samples obtained after kaolin-enhanced clotting. They were up-to 4 times higher compared with those in serum^(-) ^samples collected without clot activator and about 15 times higher than those in citrate plasma. In contrast, the MMP-2 concentrations were influenced to a less extent by the blood collection procedures (Figure 1B); in fact, in citrate plasma, MMP-2 concentrations were about 10% lower than in the other three kinds of samples. For these reasons and according to literature, plasma sample (*e.g*., obtained with citrate as anticoagulant) has been suggested to be the sample of choice for measuring circulating MMP-9 [16-18].

These data underline that MMP-9 concentrations detected in serum do not correspond to the true concentrations of MMP-9 circulating in blood. In fact, it has been demonstrated that increased MMP-9 concentrations in serum, in comparison to plasma samples, arise from the secretion of MMP-9 linked to platelet and leukocyte degranulation during coagulation/fibrinolysis processes (epiphenomenon greatly enhanced by kaolin-granulate) [8,13]. It is noteworthy to highlight that both mRNA and protein of *ABCC6*, causative of the pseudoxanthoma elasticum, have been identified in leukocytes, macrophages, and lymphocytes [19,20], and that all these white blood cells abundantly contain MMP-9 [21,22].

Preconditions for a reasonably feasible extrapolation from serum to plasma data would be based on strong correlations between serum and plasma values and equal ratios of serum to plasma values in controls and the diseased cohort (e.g., equal slopes in the regression equations between the two kinds of samples in controls and the diseased patients). Although correlations of MMP-9 and MMP-2 between serum and plasma samples exist in patients with gestational hypertension and periodontal disease [23] (but they are obviously unknown for pseudoxanthoma elasticum patients), comparative measurements in other patient groups [24] showed that the high unspecific "background" concentration of MMP-9 in serum obviously was not related to the true pathological process of interest, thus impairing the potential diagnostic performance of MMP-9 biochemical evaluation [24]. Moreover, the use of serum collected under similar conditions both in healthy and diseased patients is surely not suited to circumvent that misleading procedure especially since the technical details of sampling procedures (e.g., the presence of clot activator in serum tube, the time among sample collection, centrifugation and assay [11]) were not clearly described by Diekman et al. [1]. Furthermore, the potential difference in leukocyte counts and profiles between patients affected by pseudoxanthoma elasticum and healthy subjects may significantly affect the release from white blood cells and platelets during clotting and subsequent recovery in serum of MMP-9. These preanalytical pitfalls can be avoided by the use of more standardized conditions and the use of plasma samples [15,16]. Thus, if this known "bedside" experience of clinical scientists concerning preanalytical issues of blood sampling as important part of a study design is disregarded, it cannot be expected that serum MMPs result in reliable surrogate biomarkers [1].

## Conclusion

In conclusion, to ensure an effective translation between basic biomedical research and clinical practice, appropriate preanalytical procedures of sample collection and handling have to be laid down; this is particularly true when investigating the potential diagnostic power of a biomarker in clinical trials [25,26]. This principle should be considered already in an early stage to transfer basic science discoveries into new clinical tools. In addition, molecular medicine journals should recognize and support with their publication policy that important, though neglected issue. In particular, studies on MMPs and their tissue inhibitors TIMPs in physio-pathological conditions should address these preanalytical effects to avoid pitfalls and misinterpretations due to crucial interfering factors of blood processing, not properly taken into consideration [11].

## Competing interests

The authors declare that they have no competing interests.

## Authors' contributions

KJ had the idea and was responsible for drafting the manuscript, FM and LM contributed to the writing and critical revision of the manuscript. All authors read and approved the manuscript.
